# Molecular imaging of fibroblast activation protein after myocardial infarction using the novel radiotracer [^68^Ga]MHLL1

**DOI:** 10.7150/thno.51419

**Published:** 2021-06-22

**Authors:** Laura B.N. Langer, Annika Hess, Zekiye Korkmaz, Jochen Tillmanns, Laura M. Reffert, Jens P. Bankstahl, Frank M. Bengel, James T. Thackeray, Tobias L. Ross

**Affiliations:** 1 Department of Nuclear Medicine, Hannover Medical School; 2 Department of Cardiology and Angiology, Hannover Medical School; 3 Molecular Imaging and Radiochemistry, Department of Clinical Radiology and Nuclear Medicine, Medical Faculty Mannheim of Heidelberg University

## Abstract

**Background:** Myocardial infarction (MI) evokes an organized remodeling process characterized by the activation and transdifferentiation of quiescent cardiac fibroblasts to generate a stable collagen rich scar. Early fibroblast activation may be amenable to targeted therapy, but is challenging to identify *in vivo*. We aimed to non-invasively image active fibrosis by targeting the fibroblast activation protein (FAP) expressed by activated (myo)fibroblasts, using a novel positron emission tomography (PET) radioligand [^68^Ga]MHLL1 after acute MI.

**Methods:** One-step chemical synthesis and manual as well as module-based radiolabeling yielded [^68^Ga]MHLL1. Binding characteristics were evaluated in murine and human FAP-transfected cells, and stability tested in human serum. Biodistribution in healthy animals was interrogated by dynamic PET imaging, and metabolites were measured in blood and urine. The temporal pattern of FAP expression was determined by serial PET imaging at 7 d and 21 d after coronary artery ligation in mice as percent injected dose per gram (%ID/g). PET measurements were validated by *ex vivo* autoradiography and immunostaining for FAP and inflammatory macrophages.

**Results:** [^68^Ga]MHLL1 displayed specific uptake in murine and human FAP-positive cells (p = 0.0208). In healthy mice the tracer exhibited favorable imaging characteristics, with low blood pool retention and dominantly renal clearance. At 7 d after coronary artery ligation, [^68^Ga]MHLL1 uptake was elevated in the infarct relative to the non-infarcted remote myocardium (1.3 ± 0.3 *vs.* 1.0 ± 0.2 %ID/g, p < 0.001) which persisted to 21 d after MI (1.3 ± 0.4 *vs.* 1.1 ± 0.4 %ID/g, p = 0.013). Excess unlabeled compound blocked tracer accumulation in both infarct and non-infarct remote myocardium regions (p < 0.001). Autoradiography and histology confirmed the regional uptake of [^68^Ga]MHLL1 in the infarct and especially border zone regions, as identified by Masson trichrome collagen staining. Immunostaining further delineated persistent FAP expression at 7 d and 21 d post-MI in the border zone, consistent with tracer distribution *in vivo*.

**Conclusion:** The simplified synthesis of [^68^Ga]MHLL1 bears promise for non-invasive characterization of fibroblast activation protein early in remodeling after MI.

## Introduction

Cardiovascular disease (CVD), particularly myocardial infarction (MI) and chronic heart failure (HF), remain a leading cause of worldwide mortality 1,2. Primary therapy after myocardial infarction aims to restore blood flow to the damaged region, which improves acute survival, but long term outcome is less dependent on reperfusion alone. Chronic remodeling including matrix reorganization and active fibrosis are important components in healing, culminating in a stable scar of limited size 3-5. These processes are mediated in part by activated cardiac fibroblasts, which respond to acute inflammation to promote (myo)fibroblast transdifferentiation and scar formation. The activity of (myo)fibroblasts directly influences adverse remodeling and heightened activity is associated with worse prognosis in patients 3.

Activated cardiac (myo)fibroblasts are important mediators of the inflammatory response to myocardial ischemia by secreting chemokines, cytokines, and growth factors 6-8. However, conventional diagnostics do not effectively identify (myo)fibroblast activity, as tissue biopsies are difficult to obtain and may be subject to sampling errors due to regional heterogeneity. The membrane bound serine protease fibroblast activation protein (FAP) is expressed on the surface of activated (myo)fibroblasts and thought to be involved in ventricular remodeling by stimulating fibrosis and extracellular matrix remodeling via digestion of collagen I 9-11. Specifically, FAP is highly expressed in the first week after myocardial infarction, during the formation of stable scar 12. This involvement in remodeling may represent a valuable biomarker and therapeutic target of (myo)fibroblasts 13.

Positron emission tomography (PET) imaging of fibroblasts is gaining interest in the oncology community, with recent advent of FAP-targeted radiotracers, a versatile theranostic in multiple cancers 14-16. Since fibroblasts comprise up to 50% of the cellular content of the heart and are critical mediators of ventricular remodeling, non-invasive imaging of fibroblast activity after myocardial injury may provide crucial insights into pathogenesis and response to fibroblast-targeted therapy 17. While FAP imaging has recently been established in oncology, wider application in cardiology is only beginning to emerge. In an initial study targeted imaging identified transiently increased FAP expression in the infarct territory at 7 d after myocardial infarction in rats 18. In studying FAP expression levels after MI, non-invasive imaging of activated (myo)fibroblasts was possible.

We hypothesized that FAP-targeted PET imaging could non-invasively track dynamic fibrosis after MI in mice, providing an index of ventricular remodeling. To this end, we developed the novel FAP-targeted radiotracer [^68^Ga]MHLL1 with a simplified one-step-synthesis accessible precursor and efficient standard ^68^Ga-labeling, and evaluated its binding characteristics *in vitro* in human and murine cells 19. We further applied [^68^Ga]MHLL1 for *in vivo* PET imaging in a mouse model of MI to validate its utility to detect (myo)fibroblast activation in response to cardiac injury.

## Materials and Methods

### Materials

Chemicals were acquired from ChemShuttle, CheMatech, Sigma-Aldrich, Acros Organics, Fluka, VWR Chemicals, Merck, Cayman Chemicals, and Biochrom. Cell culture reagents were purchased from Sigma-Aldrich and Gibco. Module-based radiolabeling was conducted using the GRP-module 3V (Scintomics) including synthesis cassettes (ABX/Scintomics) and a ^68^Ge/^68^Ga-radionuclide generator (ITG Garching or IRE ELiT). Analysis was performed by high performance liquid chromatography (HPLC) on a LaChrom L7000 system (Hitachi-Merck) equipped with a Luna (C18(2)) column (Phenomenex).

### Chemical synthesis and radiolabeling

#### Radiolabeling precursor

(S)-Glyclypyrrolidine-2-carbonitrile tosylate (40 mg, 0.12 mmol) and the chelator p-benzyl-NCS-NODAGA (64.1 mg, 0.12 mmol,) were allowed to react for 72 hours in 1.3 mL 1,3-dimethylformamide (DMF). The solvent was removed under reduced pressure. The crude product was purified twice by semipreperative HPLC to yield 2,2'-(7-(1-carboxy-4-((4-(3-(2-((S)-2-cyano-pyrrolidin-1-yl)-2-oxoethyl)thio-ureido)benzyl)amino)-4-oxobutyl)-1,4,7-triazonane-1,4-diyl)diacetic acid (MHLL1) after lyophilisation as white powder (16.67 mg, 0.025 mmol, 21% yield). Analytics are provided in the supplement.

#### Non-radioactive reference compound

To obtain the *cold* reference compound, the precursor MHLL1 (30 mg, 0.045 mmol) was dissolved in 2.0 mL 1.5M HEPES buffer and stirred under ice-cooling. Ice cold 0.5 M gallium(III) chloride solution in pentane (90 μL; 0.045 mmol)) was added slowly. The reaction was allowed to warm to room temperature and heated to 100 °C for 40 min, successively. Purification was performed by SPE cartridge chromatography (SepPak plus ^t^C18 light, Waters AG) using 30% ethanol in water as eluent. The pure product, [^nat^Ga]MHLL1, was obtained in 60% yield (referred to [^nat^Ga]gallium(III) chloride). Analytics are provided in the supplement.

#### Radiolabeling

Manual radiolabeling was performed using 300 - 500 MBq (8.1 - 13.5 mCi) gallium-68 in 1.1 ml 0.1M HCl solution, derived from the Galli Ad (IRE ELiT, Belgium) radionuclide generator without post-processing, followed by addition of 37 nmol (25 µg) precursor in 1.5 M HEPES buffer and radiolabeling at 100 °C for 5 - 7 min. [^68^Ga]MHLL1 was isolated by SPE cartridge purification (SepPak plus ^t^C18 light, Waters AG), and formulated in in 5% ethanol in saline.

Automated radiosynthesis using 60 nmol precursor was performed on a GRP Module 3V (Scintomics) in combination with a ^68^Ge/^68^Ga-radionuclide generator (ITG Garching) using cationic post processing and a labeling time of 20 min at 100 °C in 1.5 M HEPES buffer. Final formulation of the tracer was in 5% ethanol in saline.

### Determination of distribution coefficient and stability

The distribution coefficient logD_pH 7.4_ was determined by the shake flask method. 250 kBq (6.7 µCi) of [^68^Ga]MHLL1 was added to a 2 mL reaction tube containing 700 µL n-octanol and 700 µL 0.01 M phosphate buffered saline (PBS, pH = 7.4). The tube was shaken (Vortexer) for 2 min and centrifuged at 12000 rpm for 5 min. The phases were drawn completely and measured in a gamma counter (2470 Wizard, Perkin Elmer). The coefficient was calculated with the formula:


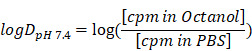


Blood was obtained from healthy volunteers for testing of tracer stability *in vitro*. To assess the stability *in vitro*, 1 MBq (0.03 mCi) [^68^Ga]MHLL1 was added to either 300 µL human serum or 300 µL PBS in an 1.5 mL reaction vessel. The tubes were held at 37 °C for defined time points. At 0, 30, 60 and 120 minutes, samples were drawn and analyzed by HPLC. Each time point was determined in triplicate.

### In vitro assays

*In vitro* uptake experiments were conducted using wildtype HT1080 cells and transfected HT1080 cells overexpressing murine FAP or human FAP 20. All cells were maintained under RMPI1640 medium supplemented with 10% fetal calf serum and 1% penicillin/streptomycin. To stabilize FAP-transfected cells, geneticin sulfate (G418, 250 µg/ml) was added every 3-5days. Cells were cultivated in T75 cell flask and incubated at 37 °C in 5% CO_2_ humidified atmosphere. One day before cell uptake assays, cells were seeded on 24 well plates at 10^5^/well. Medium was replaced 30 min prior to the assay start with HBSS buffer.

To investigate tracer binding kinetics, [^68^Ga]MHLL1 (total concentration 100 kBq/mL; 2.7 µCi/mL) was added and incubated for 1, 5, 7.5, 15, 30, 60 and 120 min at 37 °C in 5% CO_2_ humidified atmosphere. To terminate binding, supernatant was removed and cells were washed twice with ice-cold phosphate buffered saline. Membrane-bound tracer was detached by incubation in 1 mL acidic glycine (pH = 2.5) for 10 min. To delineate internalized tracer, washed cells were lysed in 200 µL of 0.5% sodium dodecyl sulfate (SDS) for 5 min. Both membrane-bound glycine and internalized SDS fractions were counted for activity in the gamma counter (2470 Wizard, Perkin Elmer).

All results were obtained in triplicates. Protein content of the lyzed cells was analyzed via Pierce™ BCA Protein Assay Kit.

To determine tracer efflux, [^68^Ga]MHLL1 (100 kBq/mL; 2.7 µCi/mL) was added to cells (~200000 per well) and incubated for 30 min, followed by washing twice with chilled PBS. Cell medium RMPI 1640 was added and incubated at 37 °C in 5% CO_2_ humidified atmosphere for a further 1, 5, 15, 30, 60, 120 and 180 min, after which supernatant was removed. Retained activity was determined by lysis with 0.5% SDS.

Binding specificity for FAP was tested by addition of cold compound in increasing concentration (0, 5, 50, 125, 250, 500 mM and 5 and 10 µM) and tracer (100 kBq/ml; 2.7 µCi/mL) with uptake over 30 min at 37 °C. Supernatant was then removed, cells washed twice with cold PBS, and membrane-bound and internalized tracer was determined by acidic glycine and 0.5% SDS lysis. The IC_50_ was calculated using the normalized internalized activity (GraphPad Prism v7.0).

### Animals

Male C57Bl/6N mice (n = 25) were purchased from Charles River and housed in groups with food and water freely available. All experiments are in accordance with the guidelines of the European Directive 2013/63/EU as well as German national laws and are approved by the local authorities and the institutional animal care and use committee.

### Surgery

Mice underwent surgical myocardial infarction (n = 14) or no surgery (n = 6), as previously described 21. Briefly, following pretreatment with butorphanol analgesic (2 mg/kg, sc), mice were anesthetized with isoflurane (induction at 3% isoflurane, 3 L/min oxygen, maintenance 1.5%), intubated, and mechanically ventilated. The heart was exposed by thoracotomy and opening of the pericardium, and a suture was permanently secured around the left coronary artery. Infarction was confirmed by tissue blanching. The surgical wound was closed and mice recovered in a separate cage.

### Small Animal PET Imaging

Distribution of [^68^Ga]MHLL1 was initially assessed in dynamic PET scans in healthy control mice (n = 6). To determine the capability to image activated cardiac fibroblasts after myocardial infarction, additional images were acquired at 7 d and/or 21 d after MI surgery (d 7, n = 9 unblocked, n = 5 blocked; d 21, n = 6 unblocked, n = 4 blocked). These timepoints were selected based on the known expression of FAP after MI in mice 12, and according to a prior imaging study using a FAP ligand 18. All PET images were obtained using the Inveon small animal PET camera (DPET, Siemens) according to previous protocols 22. Briefly, mice were anesthetized under isoflurane (1.5%, 0.6 L/min oxygen) and positioned in pairs in the imaging bed (Minerve) with the whole body in the field of view of the PET camera. [^68^Ga]MHLL1 (12.8 ± 2.0 MBq; 0.35 ± 0.05 mCi) was injected as a 150 µL bolus via a catheter inserted into a lateral tail vein. Dynamic listmode PET images were acquired initially over 60 min, with respiration monitored continuously. As the time-activity curves displayed consistent distinction between heart and blood activity between 15 - 60 min after tracer injection and defined blood clearance in later frames, subsequent images were acquired over 10 min, between 50 - 60 min after tracer injection to increase the number of animals that could be imaged with each tracer production. At the conclusion of the [^68^Ga]MHLL1 scan, a subgroup of mice were administered [^18^F]FDG (18.0 ± 3.5 MBq; 0.5 ± 0.1 mCi, ip) to delineate myocardial contours. After 20 min of uptake under isoflurane anesthesia, a static listmode acquisition was performed over 10 min. A fast CT scan was performed subsequently for anatomic registration. Images were analyzed using Inveon Research Workplace (IRW 2.0) software. Regions of interest (ROI) were defined for infarct, remote myocardium and specified organs, using the [^18^F]FDG and CT fused images to localize the organs. A threshold-based ROI was defined from the viable myocardium signal of [^18^F]FDG, whereas the infarct territory ROI was defined in the hypometabolic anterolateral ventricle wall, and these ROIs were cross-applied to the matched [^68^Ga]MHLL1 image acquired in the same space. Time-activity curves were generated from dynamic data to compare tracer distribution. Data were further analyzed as % injected dose normalized to tissue mass (%ID/g) at 50 - 60 min after tracer injection.

### Biodistribution and metabolite analysis

At the conclusion of the final PET scan, animals were killed by cervical dislocation. [^68^Ga]MHLL1 distribution was validated by *ex vivo* biodistribution and autoradiography. For *ex vivo* biodistribution, organs (heart, lung, stomach, liver, gallbladder, bowel, muscle (*quadriceps femoris*), spleen, kidneys, testicles, blood, urine) were rapidly excised, rinsed in PBS, blotted, and transferred to pre-weighed gamma-counter tubes. Samples were counted in a gamma counter (2470 Wizard, Perkin Elmer) along with a 1% standard dilution of the injected dose to calculate %ID/g.

Urine and blood were analyzed via HPLC at 60min after tracer injection (n = 5). Blood was centrifuged at 3000 *g* to isolate serum. Plasma and urine proteins were precipitated by addition of ice-cold methanol and centrifugation at 16000 *g*. Tracer and metabolites were analyzed via HPLC.

### Autoradiography and tissue staining

For regional analysis of the heart, a subgroup of animals (d7 n = 3 unblocked, n = 1 blocked; d21 n = 3 unblocked, n = 1 blocked) underwent *ex vivo* autoradiography as described previously 23. Briefly, at the end of the PET scan (60 min after tracer injection) mice were sacrificied and hearts were rapidly excised and snap frozen under liquid nitrogen in TissueTek compound. Cryosections (10 µm) in the short axis from apex to base were immediately taken, and thaw-mounted onto charged SuperFrost Plus slides (Thermo Fisher Scientific). Sections were exposed to a phosphor screen (multisensitive) for 30 min in a light impermeable cassette. The exposure was then digitalized using a Cyclon imager, and resulting images were scaled to maximal activity.

Adjacent sections (6 µm) distal and superior to the coronary occlusion were taken for histology and immunostaining. Hematoxylin/eosin staining defined general morphology. Masson trichrome delineated collagen deposition in the infarct territory. The localized anterolateral infarct territory and non-infarcted inferoseptal region guided sampling points for quantitative autoradiography in adjacent sections. Immunostaining for CD68 (macrophages, Biolegend) and FAP (R&D Systems) were conducted as described previously 12,22. Co-fluorescence-immunostaining for CD68 and fibroblast activation protein delineated the spatial relationship between FAP and macrophages.

## Results

### Synthesis and labeling of FAP Ligand [^68^Ga]MHLL1

**Fig 1:**
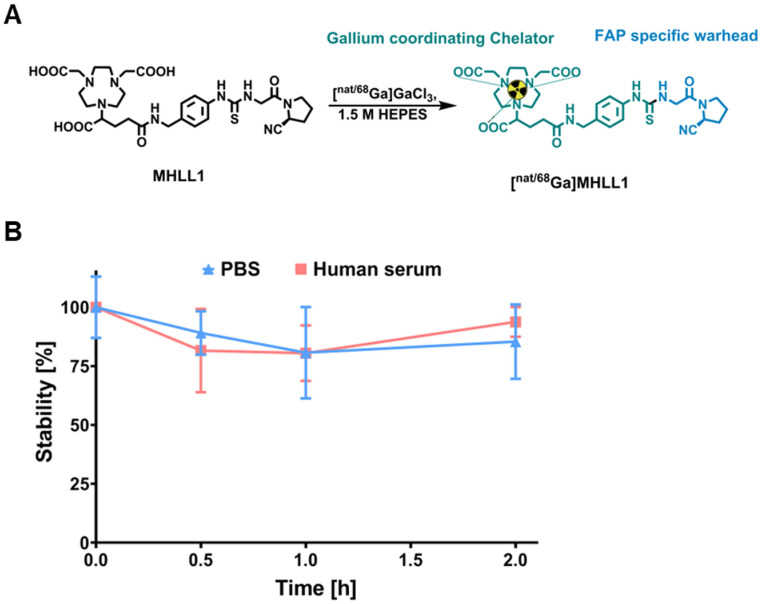
(A) Synthesis scheme of radiolabeled MHLL1 and the cold reference compound. (B) Stability of [^68^Ga]MHLL1 in PBS (pH = 7.4) and human serum (n = 4) over time (analyzed via radioHPLC).

MHLL1 was synthesized with 28% ± 16% chemical yield. The cold reference compound was obtained with 6% chemical yield (Figure 1A). NMR spectra and mass analysis confirmed the identity of the product (Figure S1-S3). Manual radiolabeling of MHLL1 provided [^68^Ga]MHLL1 in radiochemical yields of 74.4% ± 2.86% (n = 7) with an apparent molar activity of 9.68 ± 1.03 MBq/nmol, and a radiochemical purity of 71.2% ± 12.9%. First automated radiolabeling of MHLL1 resulted in radiochemical yields of 40.0% ± 5.4% with a radiochemical purity of 59.4% ± 9.3%, validated by HPLC (Figure S4).

The shelf life of the precursor was at least 5 months at -20 °C (evaluation ongoing).

### [^68^Ga]MHLL1 displays favorable chemical characteristics for *in vivo* imaging

[^68^Ga]MHLL1 was stable in PBS and human serum, as evidenced by > 80% intact tracer at 180 min (Figure 1B). At 30 min of incubation, 18% free gallium-68 was detected by HPLC, but did not increase further over time. Shake-flask octanol phase distribution demonstrated favorable hydrophilicity of [^68^Ga]MHLL1, with a logD_pH=7.4_ of ‑2.22 ± 0.45, parameters favorable for renal excretion 24.

### MHLL1 selectively accumulates in FAP-positive cell lines

**Fig 2:**
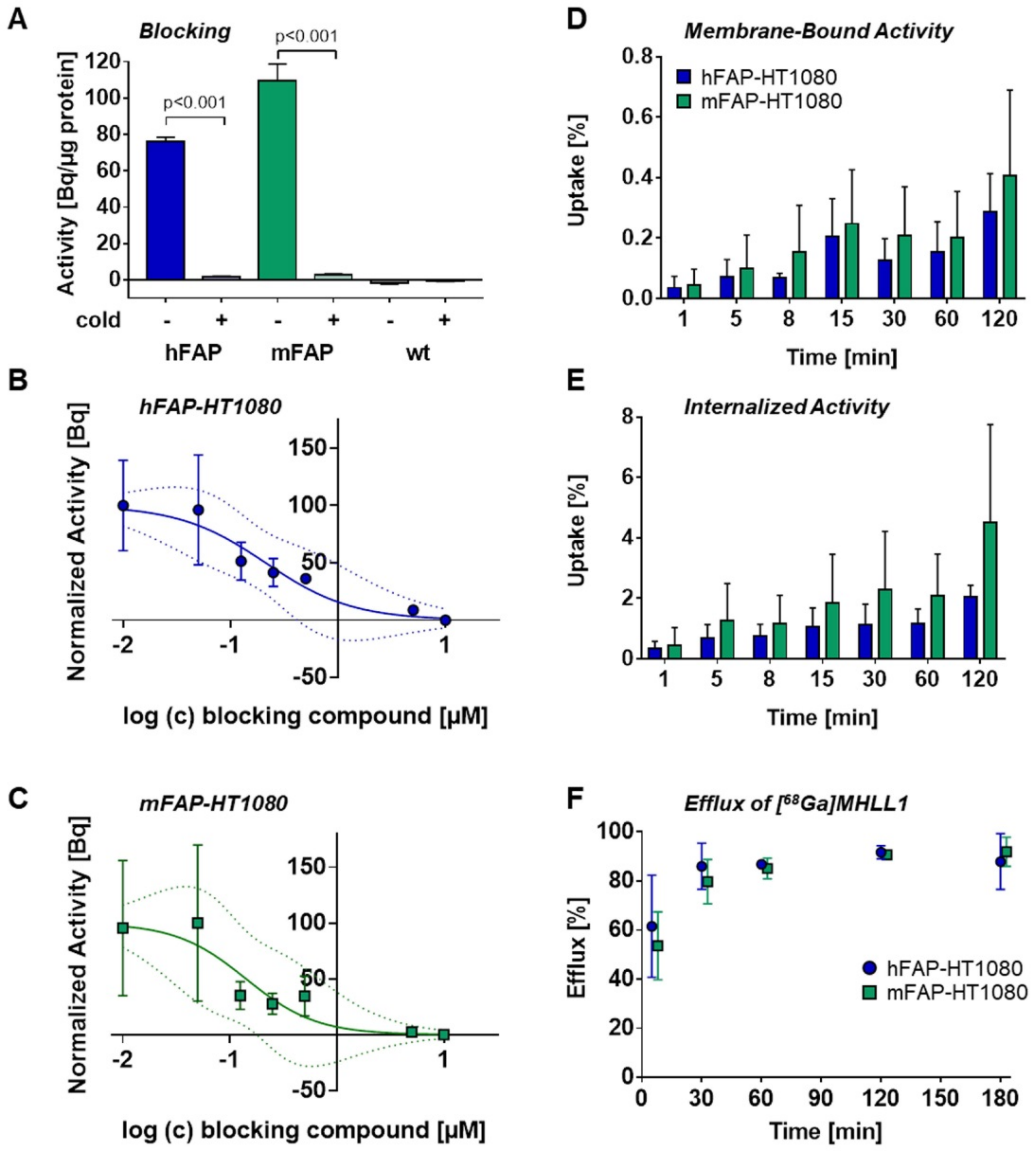
(A) *In vitro* uptake of [^68^Ga]MHLL1 in HT1080 wildtype (wt) cells and overexpressing human FAP (hFAP, blue) or murine FAP (mFAP, green) in the presence and absence of excess unlabeled (cold) reference compound. Inhibitory concentration by rising concentration of cold compound in (B) hFAP and (C) mFAP transfected HT1080 cells. **** p < 0.0001, Student's unpaired t-test. Binding kinetics assessed as (D) membrane bound, (E) internalized activity over 120 min, and (F) efflux over 180 min of tracer incubation. All measurements derived from three biological replicates in triplicate.

Uptake assays in transfected cells expressing mFAP and hFAP established specific binding of [^68^Ga]MHLL1, with markedly higher counts in FAP-expressing cells compared to wildtype negative controls (p < 0.001). Inhibition with [^nat^Ga]MHLL1 lowered tracer accumulation by 40-fold in both cell lines (Figure 2A). Displacement with the cold compound established an IC_50_ for [^68^Ga]MHLL1 of 212 nM and 142 nM for hFAP and mFAP, respectively (Figure 2B,C).

[^68^Ga]MHLL1 exhibited receptor saturation within 15 min, with rising tracer accumulation over 120 min. Internalized activity exceeded membrane-bound content by 9-11-fold in hFAP- and mFAP-transfected cells, maintained to 120 min (Figure 2D,E), suggesting rapid internalization and receptor turnover. mFAP tended to internalize more prominently than hFAP at early time points (p = 0.0208).

Efflux of [^68^Ga]MHLL1 at 30 min of incubation was 86% ± 9% in hFAP and 80% ± 9% in mFAP (Figure 2E), consistent with high turnover. Overall, < 10% of activity was retained in cells *in vitro*. HPLC confirmed efflux activity comprised intact authentic tracer (Figure S5).

### [^68^Ga]MHLL1 displays low blood pool signal and renal clearance in healthy mice

**Fig 3:**
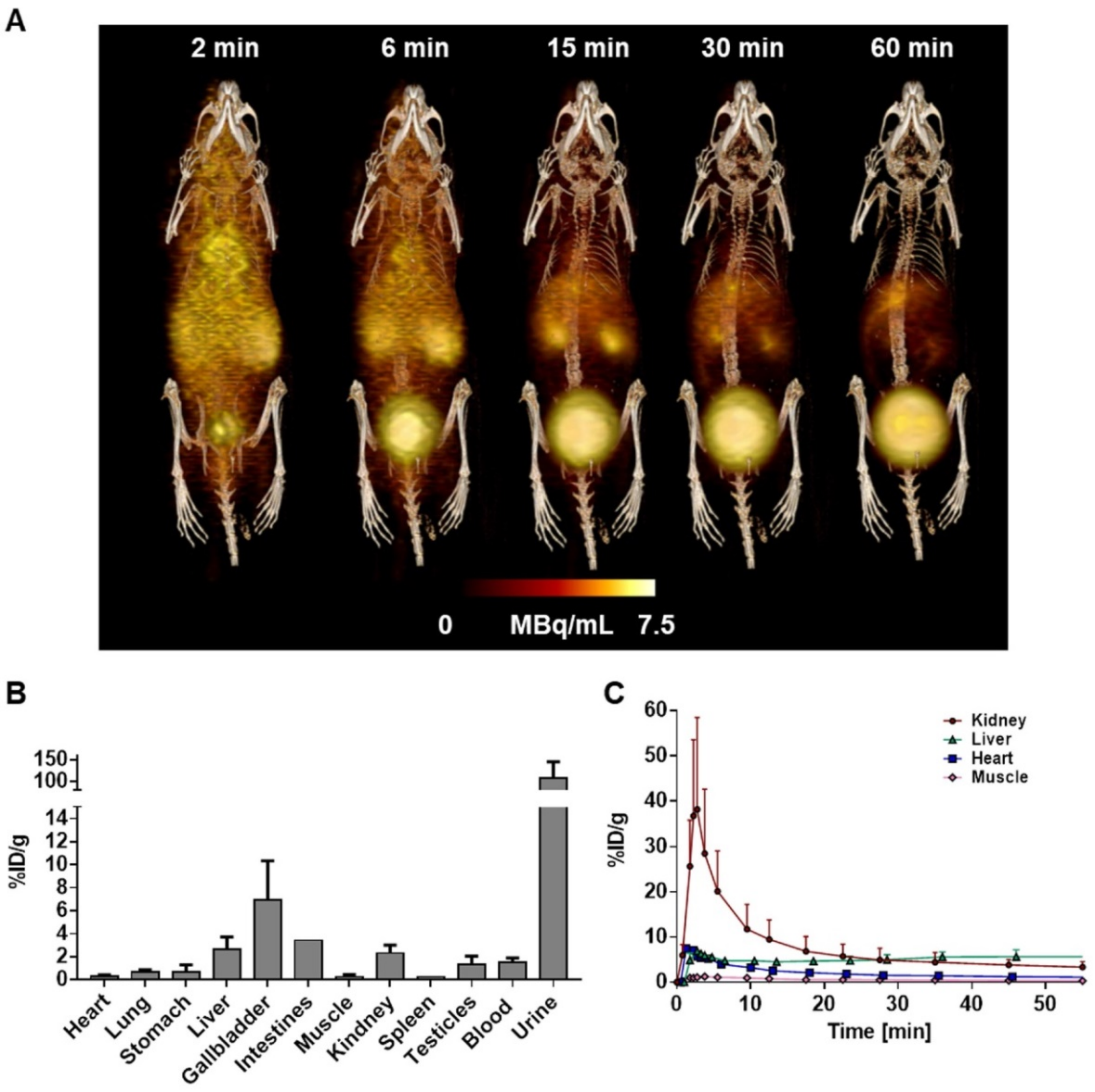
(A) Representative whole body 3D MIP PET/CT image of [^68^Ga]MHLL1 distribution in a healthy mouse at 2, 6, 15, 30, and 60 min after tracer injection displays gradual clearance from blood and accumulation in liver, kidney and urinary bladder. Distribution is paralleled by (B) *ex vivo* biodistribution counts in excised tissue at 60 min and (C) time activity curves of target organs. Mean ± SD, n = 4.

Dynamic PET scans in healthy C57BL/6 mice demonstrated rapid blood clearance and limited retention in heart, lung, and skeletal muscle (Figure 3A). Time-activity curves demonstrated highest accumulation in liver, gallbladder, and kidney, with gradually rising activity in urinary bladder, reflecting renal excretion (Figure 3C). Organ activity was validated by *ex vivo* biodistribution at 60 min (Figure 3B). HPLC metabolite analysis revealed 70.4% ± 4.3% authentic [^68^Ga]MHLL1 in blood at 60 min after tracer injection. Remaining activity included 2.1% ± 0.9% free gallium-68 and one polar metabolite (0.2% ± 0.1%) as well as two tracer impurities (28.8% ± 1.8%). In urine, authentic tracer accounted for 73.3% ± 1.8% of total activity, with two tracer impurities (17.7% ± 3.4%) and two polar metabolites (2.1% ± 0.9%) (Figure S6).

### [^68^Ga]MHLL1 specifically accumulates in the infarct territory after myocardial infarction in mice

**Fig 4:**
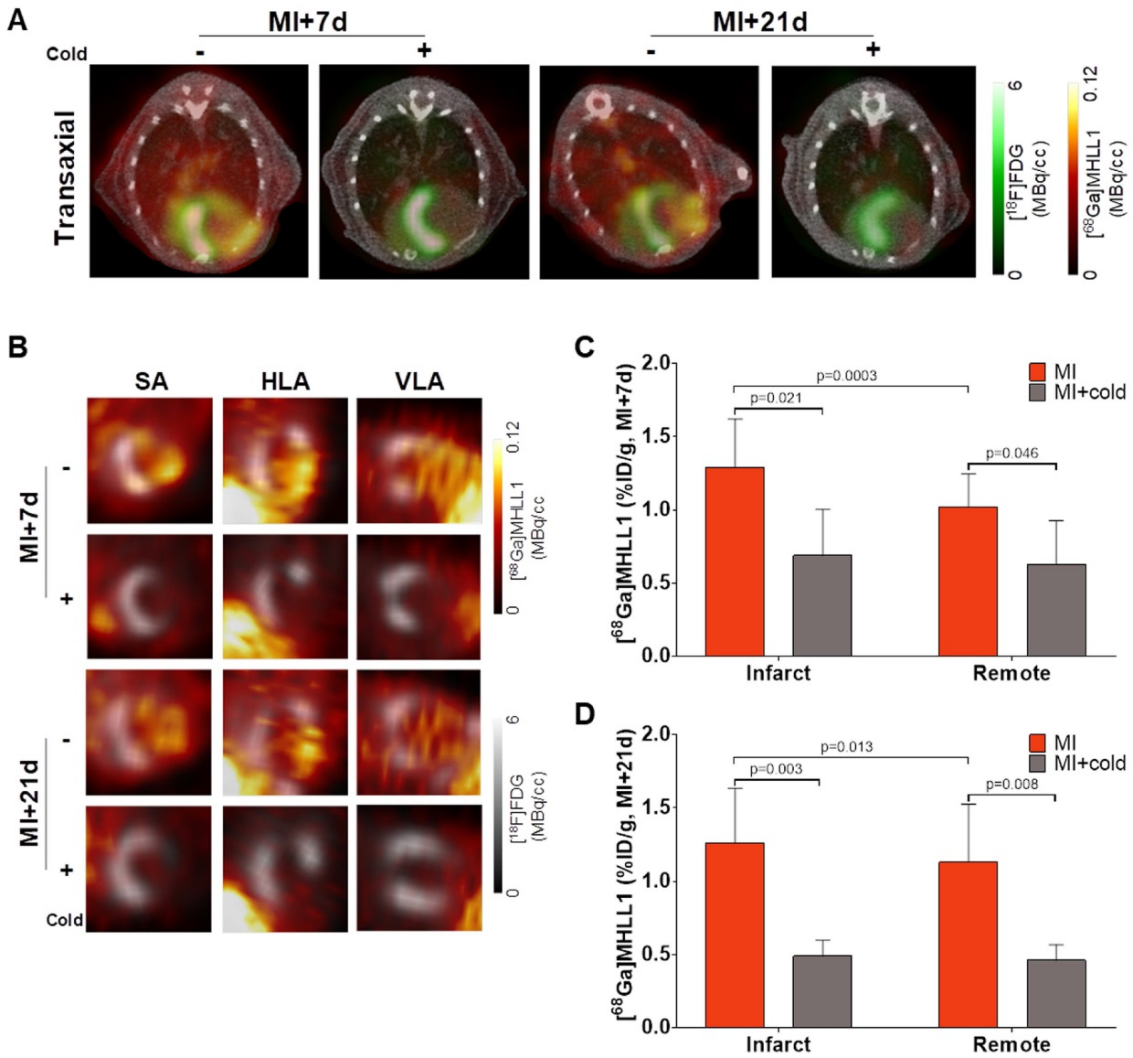
(A) Representative PET/CT fusion transaxial images at 7 d and 21 d after myocardial infarction (MI) with and without co-administration of unlabeled ligand (1mg/kg). Images display the localized accumulation of [^68^Ga]MHLL1 (orange) in the non-viable infarct territory defined by [^18^F]FDG (green) with additional distribution at the site of the surgical wound that is less prominent at 21 d after injury and limited dermal signal. (B) Reoriented cardiac axis images display the localization of the [^68^Ga]MHLL1 signal (colorscale) to the infarct defined by [^18^F]FDG (greyscale) at both timepoints, significantly lowered by blocking. Semiquantitative analysis of percent injected dose (ID) per gram in the infarct territory and non-infarcted remote myocardium shows at (C) 7 d and (D) 21 d after coronary artery occlusion confirms the selective accumulation of [^68^Ga]MHLL1 in the scar and border zone, as well as remote territory. Blocked *vs.* unblocked, one way analysis of variance, Bonferroni post hoc test; infarct *vs.* remote Students two-tailed paired t-test. Sample sizes (7 d MI n = 9, sham = 5; 21 d MI n = 6, sham n = 4).

To evaluate the suitability of [^68^Ga]MHLL1 to image fibroblast activation during ventricle remodeling in the heart, serial PET imaging was conducted at 7 d and 21 d after coronary artery occlusion in mice. *In vivo* PET/CT visualized accumulation of [^68^Ga]MHLL1 in the nonviable infarct region delineated by absence of [^18^F]FDG uptake, and modest signal emanating from the surgical wound 7 d after MI (Fig 4A,B). Co-administration of excess cold ligand (1 mg/kg (1.48 µmol/kg), iv) lowered the detected signal throughout the thoracic region, and particularly in the infarct territory. Subsequent imaging of the stable scar at 21 d after injury displayed persistently elevated specific [^68^Ga]MHLL1 uptake in the infarct region. Reorientation in the cardiac axis localizes the [^68^Ga]MHLL1 signal to the anterolateral infarct wall (Figure S7). Semi-quantitative analysis displayed higher uptake in the infarct territory compared to non-infarct remote myocardium at 7 d after MI (1.3 ± 0.3 *vs.* 1.0 ± 0.2 %ID/g, p < 0.001; Fig 4C). While this difference between infarct and non-infarct signal remained at 21 d, variability was higher and absolute difference reduced (1.3 ± 0.4 *vs.* 1.1 ± 0.4 %ID/g, p = 0.013, Fig 4D). Time-activity curves for the infarct and remote myocardium displayed modest deviation, beginning from 15 min after tracer injection, which is less distinct at 21 d after MI (Figure S8). Co-administration of cold ligand significantly lowered the accumulation of [^68^Ga]MHLL1 by 40% at 7 d and 60% at 21 d after MI (p < 0.01). By contrast, no blocking effect was observed in liver or gallbladder.

### Autoradiography and histology confirm the localization of [^68^Ga]MHLL1 binding to infarct and border zone regions

**Fig 5:**
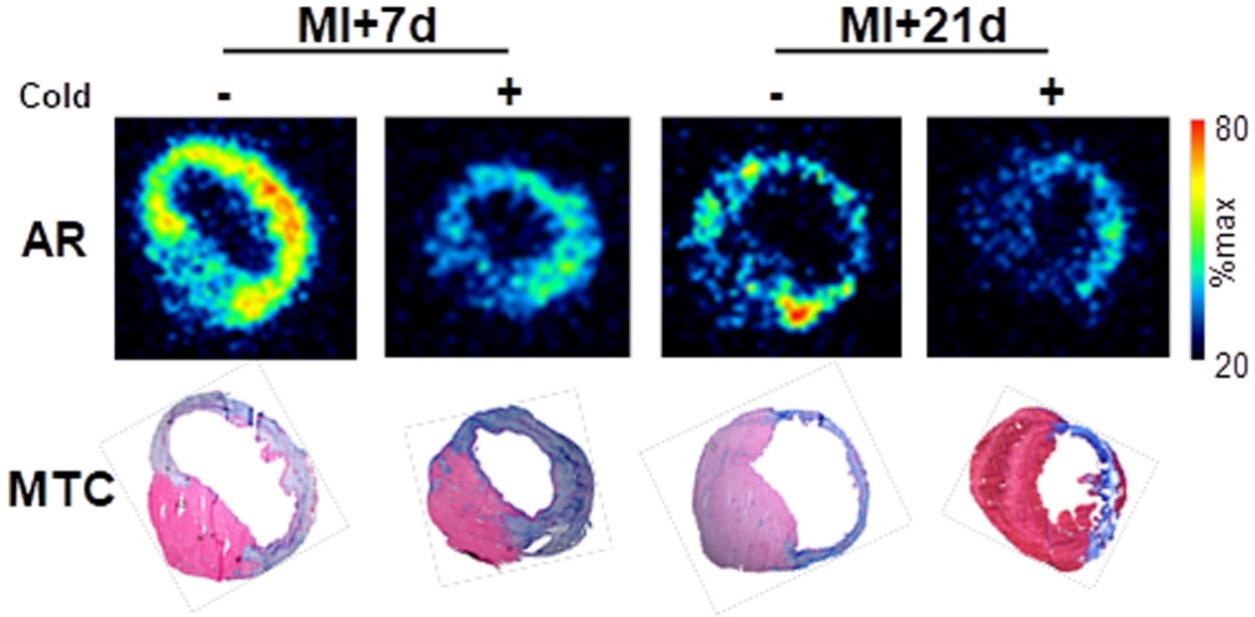
*Ex vivo* autoradiography (AR) at 7 d and 21 d after myocardial infarction (MI) and Masson trichrome histology (MTC) in adjacent sections shows the selective accumulation of [^68^Ga]MHLL1 in the infarct and border zone regions which is blocked by the co-administration of unlabeled compound (1 mg/kg). Autoradiogram images are scaled to the maximum signal for regional localization.

To confirm the localization of tracer binding independent of liver activity spillover, a subgroup of mice at each timepoint were sacrificed at the conclusion of the PET scan, and hearts were excised exposed for *ex vivo* autoradiography and histopathology. Autoradiography displayed elevated signal in the infarct and border zone territories after MI at 7 d, with a modestly lower signal 21 d after MI. Co-administration of cold compound lowered [^68^Ga]MHLL1 uptake in the infarct territory and in the remote non-infarct region (Fig 5). Alignment with Masson trichrome stained sections delineated localization of the signal to the transmural infarct region, as well as moderately elevated activity in the remote myocardium (Fig 5).

### Immunostaining demonstrates persistent FAP in the infarct and border zone over 21 d after MI

**Fig 6:**
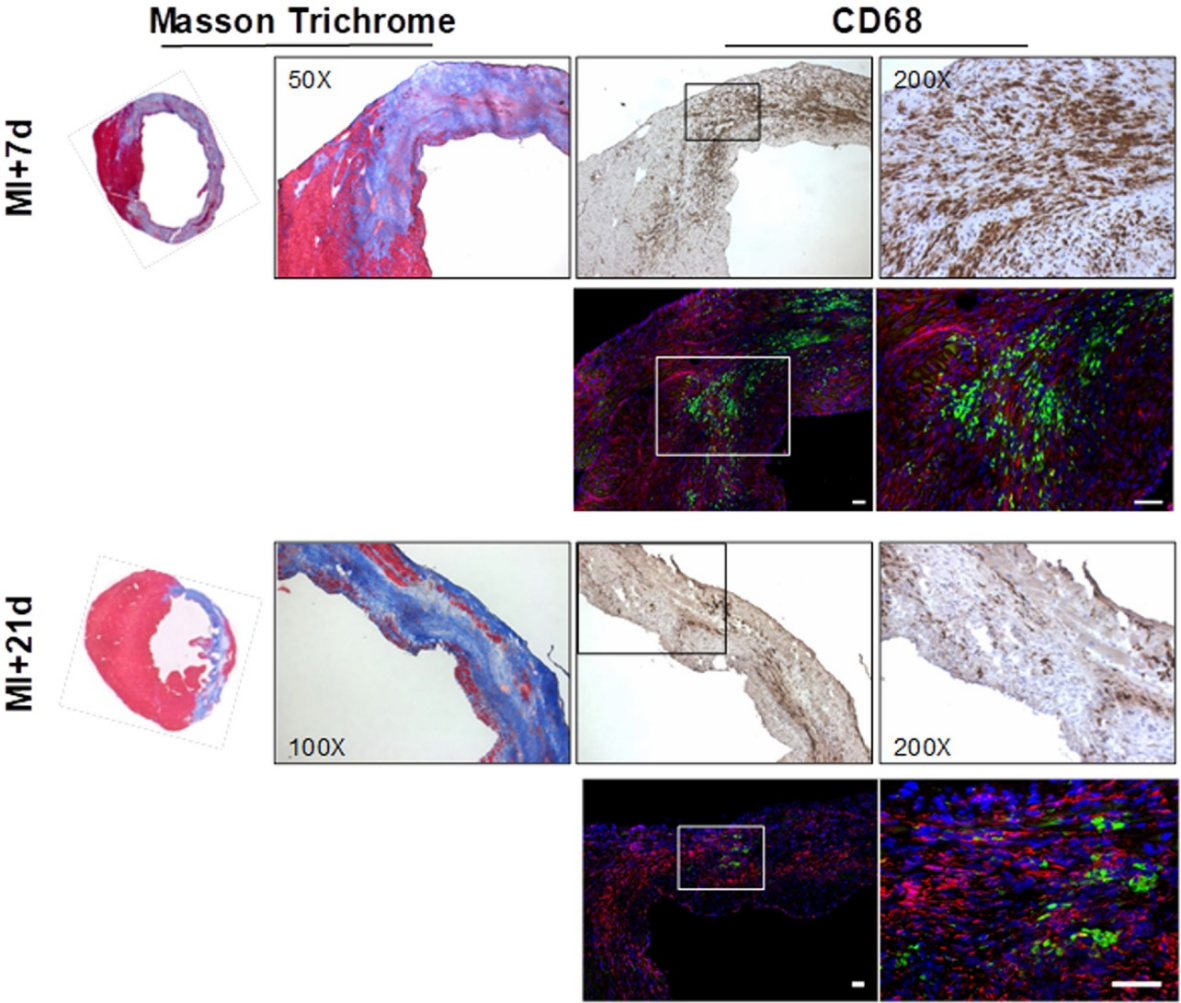
Overview Masson trichrome staining (left panels) localizes the collagen-rich scar (blue) and border zone territory at 7 d (upper panels) and 21 d (lower panels) after myocardial infarction. Immunostaining for CD68 identifies robust macrophage infiltration in the infarct region. Co-immunofluorescence staining in the same sections reveals adjacent fibroblast activation protein (FAP) staining (red) in the border zone territory at both 7 d and 21 d, in the vicinity of CD68 positive macrophages (green) at 7 d, which decline significantly by 21 d after infarction. Nuclei are stained with DAPI (blue) in fluorescence images. Scale bar = 50 µm.

Overview histology displayed the gradual development of the fibrotic scar after coronary artery occlusion. The infarct region and border zone exhibit high levels of CD68-positive macrophages at 7 d after injury, which decline as the transmural scar develops at 21 d (Fig 6). Co-immunofluorescence staining identified the presence of FAP in the infarct and border zone territory at 7 d after MI with focal populations of CD68-positive macrophages in close proximity (Fig 6). Notably, while the macrophage content is decreased at 21 d, the FAP staining is persistent, consistent with the *in vivo* imaging signal.

## Discussion

Heart failure development after MI depends on the severity of remodeling, including the reorganization of extracellular matrix and replacement fibrosis. This process is mediated by cardiac fibroblast activation, which represent a potential therapeutic target 13. Conventional imaging techniques struggle to identify sites of remodeling and ongoing fibrosis. Here, we developed a novel radiotracer to noninvasively evaluate fibroblast activation after MI. The precursor MHLL1 is accessible via a simple and convenient one-step coupling reaction of commercially-available starting materials, albeit with the need of extensive HPLC purification. Subsequent radiolabeling is straightforward and provides [^68^Ga]MHLL1 in good radiochemical yield from automated module synthesis as well as manual synthesis. [^68^Ga]MHLL1 displayed selective binding to FAP-expressing cells with rapid internalization on i*n vitro* testing with limited in the infarct territory 7 d after MI, co-localized to FAP-expressing cells and active scar formation in the infarct border zone.

Extracellular matrix reorganization leading to focal or interstitial fibrosis is a common endpoint of cardiovascular disease. In the healthy heart, cardiac fibroblasts comprise 40% - 60% of total cell content, providing structural support and secreting growth factors and cytokines to maintain cardiac integrity. Following injury, cardiac fibroblasts transdifferentiate to (myo)fibroblasts, which are chemotactically recruited to the damaged region, where they secrete cytokines that contribute to inflammation 17. The ubiquitous and early involvement of fibroblasts in cardiac pathology, and their prevalence at the injury site, has precipitated interest in targeting fibroblasts to facilitate tissue repair by modulating recruitment and activity 12,13 or reprogramming fibroblasts to generate cardiomyocytes 25,26. Numerous cardiovascular drugs have ancillary anti-fibrotic benefits 13. Accordingly, non-invasive methods to detect and track fibroblast activation are attractive.

After cardiac injury, (myo)fibroblasts strongly express FAP, though its physiologic role remains unclear 27. Early tissue repair requires regulated fibrinolysis, where proteolytic activity of proteases including FAP is critical. FAP also enables fibroblast migration and facilitates (myo)fibroblasts infiltration to damaged tissue 28. This transient enrichment renders FAP an optimal imaging biomarker of fibroblast activation. In the present study, the novel radiotracer [^68^Ga]MHLL1 displays specific uptake in murine and human FAP transfected HT1080 cells. We also observed preferential internalization of bound tracer over surface binding, in agreement with earlier findings 16,29. Nonetheless, bound tracer is also rapidly exported, consistent with rapid kinetics of FAP.^15^,^30^

Optimal molecular imaging agents exhibit limited blood retention and low background signal, affording high contrast in the target organ(s) 31,32. [^68^Ga]MHLL1 displayed rapid blood clearance and predominant renal clearance, giving high image contrast at 50 - 60 min after tracer injection. *In vivo* PET imaging in healthy and MI mice demonstrated rapid washout of [^68^Ga]MHLL1 from all organs, predominant renal clearance, and low blood pool activity. After MI, [^68^Ga]MHLL1 detected sites of ongoing fibrosis with high tracer accumulation and contrast at 7 d during active fibrosis, consistent with the reported time course of FAP expression in the first week after ischemic injury 12.

Because the infarct territory and border zone comprise several cell populations, we cannot definitively identify the cellular basis of the [^68^Ga]MHLL1 signal. However, one benefit of FAP as an imaging target is the low expression by other cell types 18. We have shown no co-localization of FAP and CD68 macrophages on immunostaining, and the regional localization is consistent with activated fibroblasts 12. While detailed tissue analysis may further characterize the cellular basis of the imaging signal, we are reasonably confident that FAP-specific imaging identifies cardiac fibroblasts.

FAP imaging may monitor extracellular matrix reorganization and scar generation after MI, wherein it is robustly expressed during chronic inflammation and fibrosis. While tissue expression of FAP rises, serum concentration declines, inversely proportional to the severity of fibrosis in liver disease and acute coronary syndrome 33,34. This balance between fibroblast-bound and serum FAP may be exploited as a diagnostic and prognostic biomarker of disease severity.

Several FAP-targeted tracers have been developed, including antibody and small peptide constructs with demonstrable selective binding to FAP 28,35. More recently, small molecule tracers including iodine-125-MIP-1232 and gallium-68-FAPI-02 ,-04 and -13 have been validated. Iodine-125-MIP-1232 exhibited moderate affinity and low specificity in human carotid plaques and xenograft tissue 36. Alternatively, highly selective FAP tracers gallium-68-FAPI-02, -04 and -13 display high specificity and have been effectively deployed for *theranostic* treatment of cancer patients 14-16,37. Compared to the ^68^Ga-FAPI series, chemical synthesis for [^68^Ga]MHLL1 is simplified, potentially enabling faster (chemical) access to a FAP-specific radiotracer. Conversely, the presence of two major precursor side products may complicate the interpretation of images and contribute to non-specific accumulation of the tracer. While this may be rectified by more stringent precursor purification, dedicated analysis of tracer stability in the infarct territory and direct comparison to other FAP ligands would be advisable.

Recently, [^68^Ga]FAPI-04 was reported to accumulate in the infarct region with high contrast 6 d after MI in rats. This signal declined by 14 d, suggesting identification of activated fibroblasts during active remodeling 18. By contrast, [^68^Ga]MHLL1 displays sustained signal at 7 d and 21 d after MI, with signal emanating not only from the infarct territory, but also from the remote non-infarcted myocardium. This observation may indicate sensitivity of [^68^Ga]MHLL1 to both replacement scar fibrosis and reactive interstitial fibrosis. Notably, unlike the previous study, immunostaining at 21 d confirmed persistent expression of FAP which was detected by our tracer. [^68^Ga]FAPI-04 also exhibited significant uptake in the skin, suggesting uptake by dermal fibroblasts. While uptake at the surgical wound site is evident in [^68^Ga]MHLL1 images, the absolute tracer accumulation in the skin is lower than observed for [^68^Ga]FAPI-04. This discrepancy may reflect species differences between mouse and rat FAP isoforms. Direct comparisons between the two tracers in identical models may provide further insights into this distinct kinetics.

### Limitations

Some limitations should be considered in the present study. First, *in vivo* imaging was restricted to two time points after MI based on the timecourse of FAP expression described in mice and the uptake pattern of other FAP-targeted radiotracers. We were primarily interested in whether the infarct territory signal could be detected. Histopathology confirmed the expression of FAP in this timeframe. Future studies should evaluate the significance of this signal for prognosis and therapeutic monitoring.

Second, we have restricted semi-quantitative tracer uptake analysis to %ID/g rather than applying compartmental modeling. Since FAP is rapidly turned over at the cell surface of activated fibroblasts, kinetic analysis could provide added value to interpret differences in specific binding of [^68^Ga]MHLL1 between the infarct and non-infarcted remote myocardium signal. Such approaches may be better served with longer lived radioisotopes, and ^18^F-labeled FAP ligands would be desirable. Further studies into kinetic behaviour of [^68^Ga]MHLL1 and other FAP ligands are warranted.

Third, we cannot exclude the contribution of partial volume effects on the quantification of the image signal, particularly in the infarct wall which exhibits significant thinning at 21 d and is adjacent to high activity in the liver. However, complementary evaluation of *ex vivo* autoradiography indicates specificity of the [^68^Ga]MHLL1 infarct signal, co-localized to both fibrotic tissue and FAP.

Fourth, the MHLL1 precursor revealed challenging in HPLC purification which resulted in moderate radiochemical purities after labeling. In semi-preparative HPLC purification runs, quantitative removal of two major side products from the precursor was not possible. Further optimization while maintaining the one-step approach, should improve purity. An automated radiolabeling test of [^68^Ga]MHLL1 was successful, but still needs further optimization.

## Conclusions

The novel one-step synthesis radiotracer [^68^Ga]MHLL1 displays selective binding to FAP in the infarct territory and border zone, as well as remote non-infarcted myocardium, after MI. Molecular imaging of FAP expression may identify early stages of myocardial replacement and reactive fibrosis, which bears potential to guide new therapies targeting fibroblast activation.

## Abbreviations

[^18^F]FDG: 2-[18F]fluoro-2-deoxy-D-glucose
[^68^Ga]MHLL1: [^68^Ga]Ga-2,2'-(7-(1-carboxy-4-((4-(3-(2-((S)-2-cyano-pyrrolidin-1-yl)-2-oxoethyl)thioureido)benzyl)amino)-4-oxobutyl)-1,4,7-triazonane-1,4-diyl)diacetic acid
CVD: cardiovascular disease
DPP4: dipetidyl petidase-4
FAP: fibroblast activation protein
HF: heart failure
MI: myocardial infarction
PET: positron emission tomography
ROI: region of interest
SA: short axis
VLA: vertical long axis

## References

[1] WHO. Cardiovascular diseases. 2017.

[2] Heidenreich PA, Albert NM, Allen LA (2013). Forecasting the impact of heart failure in the united states a policy statement from the american heart association. Circ Heart Fail.

[3] O'Hanlon R, Grasso A, Roughton M (2010). Prognostic significance of myocardial fibrosis in hypertrophic cardiomyopathy. J Am Coll Cardiol.

[4] Kwon DH, Smedira NG, Rodriguez ER (2009). Cardiac magnetic resonance detection of myocardial scarring in hypertrophic cardiomyopathy: correlation with histopathology and prevalence of ventricular tachycardia. J Am Coll Cardiol.

[5] Weber KT, Janicki JS, Shroff SG, Pick R, Chen RM, Bashey RI (1988). Collagen remodeling of the pressure-overloaded, hypertrophied nonhuman primate myocardium. Circ Res.

[6] Virag JI, Murry CE (2003). Myofibroblast and endothelial cell proliferation during murine myocardial infarct repair. Am J Pathol.

[7] Sun Y, Weber KT (1996). Angiotensin converting enzyme and myofibroblasts during tissue repair in the rat heart. J Mol Cell Cardiol.

[8] Turner NA, Das A, Warburton P, O'Regan DJ, Ball SG, Porter KE (2009). Interleukin-1α stimulates proinflammatory cytokine expression in human cardiac myofibroblasts. Am J Physiol Circ Physiol.

[9] Niedermeyer J, Kriz M, Hilberg F (2000). Targeted disruption of mouse fibroblast activation protein. Mol Cell Biol.

[10] Rettig WJ, Garin-Chesa P, Healey JH (1993). Regulation and heteromeric structure of the fibroblast activation protein in normal and transformed cells of mesenchymal and neuroectodermal origin. Cancer Res.

[11] Park JE, Lenter MC, Zimmermann RN, Garin-Chesa P, Old LJ, Rettig WJ (1999). Fibroblast activation protein, a dual specificity serine protease expressed in reactive human tumor stromal fibroblasts. J Biol Chem.

[12] Tillmanns J, Hoffmann D, Habbaba Y (2015). Fibroblast activation protein alpha expression identifies activated fibroblasts after myocardial infarction. J Mol Cell Cardiol.

[13] Travers JG, Kamal FA, Robbins J, Yutzey KE, Blaxall BC (2016). Cardiac Fibrosis: The Fibroblast Awakens. Circ Res.

[14] Giesel FL, Kratochwil C, Lindner T (2019). ^68^Ga-FAPI PET/CT: Biodistribution and preliminary dosimetry estimate of 2 DOTA-containing FAP-targeting agents in patients with various cancers. J Nucl Med.

[15] Loktev A, Lindner T, Mier W (2018). A tumor-imaging method targeting cancer-associated fibroblasts. J Nucl Med.

[16] Lindner T, Loktev A, Altmann A (2018). Development of quinoline-based theranostic ligands for the targeting of fibroblast activation protein. J Nucl Med.

[17] Baum J, Duffy HS (2011). Fibroblasts and myofibroblasts: what are we talking about?. J Cardiovasc Pharmacol.

[18] Varasteh Z, Mohanta S, Robu S (2019). Molecular imaging of fibroblast activity after myocardial infarction using a ^68^Ga-labeled fibroblast activation protein inhibitor, FAPI-04. J Nucl Med.

[19] Langer L, Hess A, Reffert LM (2019). Visualisation of fibrosis after tissue damage with PET - A tracer for the fibroblast activation protein. J Label Compd Radiopharm.

[20] Wajant H, Moosmayer D, Wüest T (2001). Differential activation of TRAIL-R1 and -2 by soluble and membrane TRAIL allows selective surface antigen-directed activation of TRAIL-R2 by a soluble TRAIL derivative. Oncogene.

[21] Thackeray JT, Hupe HC, Wang Y (2018). Myocardial inflammation predicts remodeling and neuroinflammation after myocardial infarction. J Am Coll Cardiol.

[22] Thackeray JT, Derlin T, Haghikia A (2015). Molecular imaging of the chemokine receptor CXCR4 after acute myocardial infarction. JACC Cardiovasc Imaging.

[23] Thackeray JT, Bankstahl JP, Wang Y (2015). Targeting post-infarct inflammation by PET imaging: comparison of (68)Ga-citrate and (68)Ga-DOTATATE with (18)F-FDG in a mouse model. Eur J Nucl Med Mol Imaging.

[24] Lewis JS, Windhorst AD, Zeglis BM (2019). Radiopharmaceutical Chemistry. 1st ed. Berlin, Germany: Springer International Publishing.

[25] Bektik E, Fu J (2019). Ameliorating the fibrotic remodeling of the heart through direct cardiac reprogramming. Cells.

[26] Aghajanian H, Kimura T, Rurik JG (2019). Targeting cardiac fibrosis with engineered T cells. Nature.

[27] Juillerat-Jeanneret L, Tafelmeyer P, Golshayan D (2017). Fibroblast activation protein-α in fibrogenic disorders and cancer: more than a prolyl-specific peptidase?. Expert Opin Ther Targets.

[28] Terry SYAA, Koenders MI, Franssen GM (2016). Monitoring Therapy Response of Experimental Arthritis with Radiolabeled Tracers Targeting Fibroblasts, Macrophages, or Integrin v 3. J Nucl Med.

[29] Baird SK, Allan L, Renner C, Scott FE, Scott AM (2015). Fibroblast activation protein increases metastatic potential of fibrosarcoma line HT1080 through upregulation of integrin-mediated signaling pathways. Clin Exp Metastasis.

[30] Artym V V, Kindzelskii AL, Chen W-T, Petty HR (2002). Molecular proximity of seprase and the urokinase-type plasminogen activator receptor on malignant melanoma cell membranes: dependence on beta1 integrins and the cytoskeleton. Carcinogenesis.

[31] Sharma R, Aboagye E (2011). Development of radiotracers for oncology - the interface with pharmacology. Br J Pharmacol.

[32] Zhou Y, Liu S (2011). ^64^Cu-labeled phosphonium cations as PET radiotracers for tumor imaging. Bioconjug Chem.

[33] Williams KH, Viera de Ribeiro AJ, Prakoso E (2015). Lower serum fibroblast activation protein shows promise in the exclusion of clinically significant liver fibrosis due to non-alcoholic fatty liver disease in diabetes and obesity. Diabetes Res Clin Pract.

[34] Tillmanns J, Widera C, Habbaba Y (2013). Circulating concentrations of fibroblast activation protein α in apparently healthy individuals and patients with acute coronary syndrome as assessed by sandwich ELISA. Int J Cardiol.

[35] van der Geest T, Roeleveld DM, Walgreen B (2018). Imaging fibroblast activation protein to monitor therapeutic effects of neutralizing interleukin-22 in collagen-induced arthritis. Rheumatology.

[36] Meletta R, Herde AM, Chiotellis A (2015). Evaluation of the radiolabeled boronic acid-based FAP Inhibitor MIP-1232 for atherosclerotic plaque imaging. Molecules.

[37] Kratochwil C, Flechsig P, Lindner T (2019). ^68^Ga-FAPI PET/CT: Tracer uptake in 28 different kinds of cancer. J Nucl Med.

