# Supplementary material for: Molecular imaging of fibroblast activation protein after myocardial infarction using the novel radiotracer [68Ga]MHLL1
Source: Theranostics. 2021 Jun 22;11(16):7755–66. doi: 10.7150/thno.51419 (PMC8315078; doi:10.7150/thno.51419)

Supplementary File

Article Title: Molecular Imaging of Fibroblast Activation Protein after Myocardial Infarction using the Novel Radiotracer [^{68}Ga]MHLL1

Journal Name: Theranostics

Authors: Laura B.N. Langer[a,c§], Dr. Annika Hess[a§], Zekiye Korkmaz[a], PD Dr. Jochen Tillmanns[b], Dr. Laura M. Reffert[a,c], Dr. Jens P. Bankstahl[a], Prof. Dr. Frank M. Bengel[a], PhD James T. Thackeray[a,$], and Prof. Dr. Tobias L. Ross[a,$]

[§] equally contributing first authors; [$] equally contributing senior authors

Institutional affiliations:
[a] Department of Nuclear Medicine, Hannover Medical School, Carl-Neuberg-Strasse 1, 30625 Hannover, Germany

[b] Department of Cardiology and Angiology, Hannover Medical School, Carl-Neuberg-Strasse 1, 30625 Hannover, Germany

[c] Molecular Imaging and Radiochemistry, Department of Clinical Radiology and Nuclear Medicine, Medical Faculty Mannheim of Heidelberg University, Theodor-Kutzer-Ufer 1-3, 68167 Mannheim, Germany

[$] contributed equally

Corresponding author: Prof. Dr. Tobias L. Ross, Department of Nuclear Medicine, Hannover Medical School; phone number +49 511 532-5895; fax number: +49 511 532-18547; e-mail: ross.tobias@mh-hannover.de

Figure S1: ^{1}H-NMR spectra: MHLL1

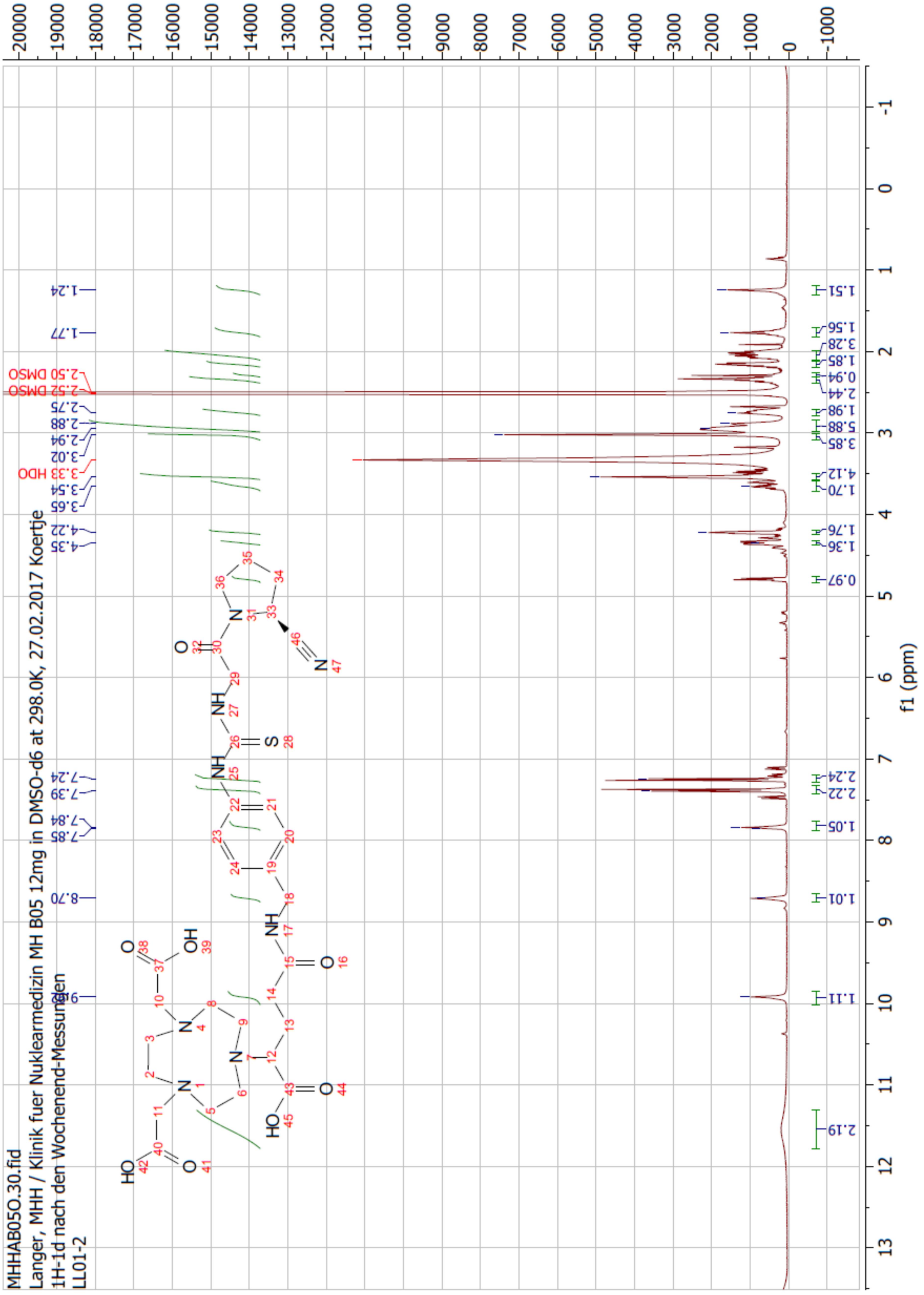

Figure S2: UPLC-MS Mass data: MHLL1

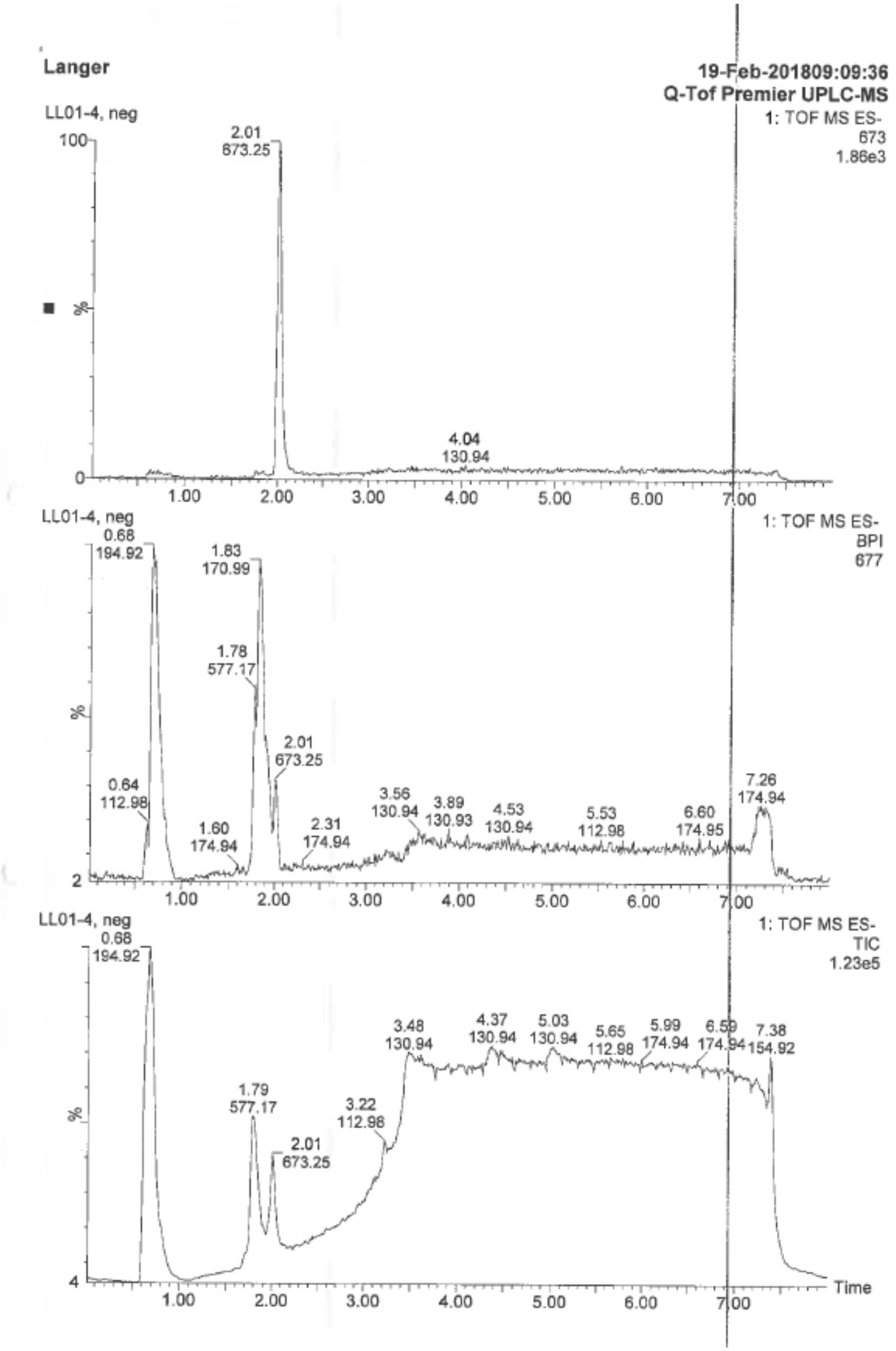

Elemental Composition Report

Single Mass Analysis

Tolerance = 10.0 PPM / DBE: min = -1.5, max = 50.0
Selected filters: None

Monoisotopic Mass, Even Electron Ions
3264 formula(e) evaluated with 43 results within limits (up to 50 closest results for each mass)
Elements Used:
C: 0-55 H: 0-80 N: 0-8 O: 0-8 Na: 0-1 S: 0-2

Langer Q-Tof Premier UPLC-MS 19-Feb-201809:09:36
LL01-4, neg 215 (2.033) **AM** (Cen,5, 65.00, Ar,11000.0,554.26,0.55,LS 5) 1: TOF MS ES- 1.34e3

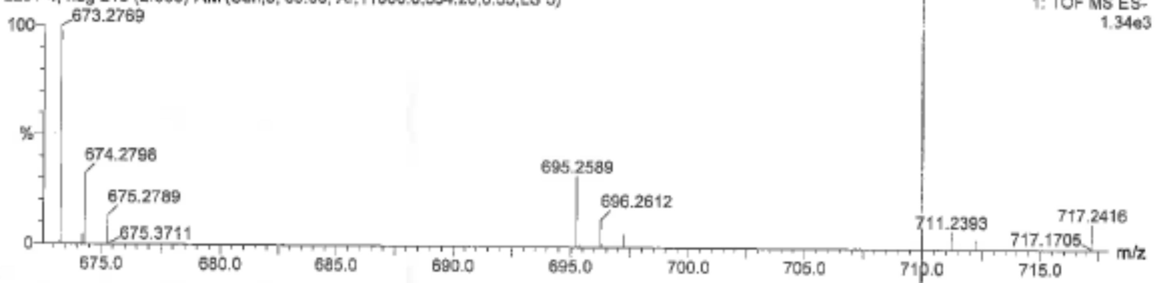

..inimum:				-1.5		
Maximum:		5.0	10.0	50.0		
Mass	Calc. Mass	mDa	PPM	DBE	i-FIT	Formula
673.2769	673.2770	-0.1	-0.1	17.5	34.4	C38 H45 N2 O5 S2
	673.2768	0.1	0.1	14.5	5.5	C30 H41 N8 O8 S
	673.2775	-0.6	-0.9	23.5	26.8	C38 H37 N6 O6
	673.2776	-0.7	-1.0	26.5	51.7	C46 H41 O3 S
	673.2777	-0.8	-1.2	19.5	26.2	C40 H42 O8 Na
	673.2778	-0.9	-1.3	6.5	4.5	C25 H46 N8 O8 Na S2
	673.2759	1.0	1.5	19.5	32.2	C37 H42 N6 O Na S2
	673.2783	-1.4	-2.1	22.5	41.2	C39 H41 N6 O S2
	673.2784	-1.5	-2.2	15.5	10.5	C33 H42 N6 O6 Na S
	673.2752	1.7	2.5	23.5	42.2	C44 H42 O3 Na S
	673.2786	-1.7	-2.5	18.5	43.4	C41 H46 O3 Na S2
	673.2751	1.8	2.7	20.5	22.5	C36 H38 N6 O6 Na
	673.2750	1.9	2.8	27.5	41.1	C42 H37 N6 O S
	673.2791	-2.2	-3.3	24.5	33.9	C41 H38 N4 O4 Na
	673.2746	2.3	3.4	14.5	26.1	C36 H46 N2 O5 Na S2
	673.2744	2.5	3.7	11.5	2.7	C28 H42 N8 O8 Na S
	673.2743	2.6	3.9	31.5	58.5	C49 H37 O3
	673.2743	2.6	3.9	18.5	24.3	C34 H41 N8 O3 S2
	673.2801	-3.2	-4.8	22.5	32.2	C42 H41 O8
	673.2736	3.3	4.9	22.5	32.9	C41 H41 N2 O5 S
	673.2802	-3.3	-4.9	9.5	7.6	C27 H45 N8 O8 S2
	673.2734	3.5	5.2	19.5	17.6	C33 H37 N8 O8
	673.2804	-3.5	-5.2	29.5	42.3	C42 H34 N8 Na
	673.2730	3.9	5.8	13.5	19.3	C33 H45 N4 O7 S2
	673.2808	-3.9	-5.8	18.5	16.1	C35 H41 N6 O6 S
	673.2810	-4.1	-6.1	21.5	53.5	C43 H45 O3 S2
	673.2811	-4.2	-6.2	14.5	16.9	C37 H46 O8 Na S
	673.2726	4.3	6.4	24.5	32.8	C40 H38 N6 O Na S
	673.2815	-4.6	-6.8	27.5	40.5	C43 H37 N4 O4
	673.2818	-4.9	-7.3	10.5	11.7	C30 H46 N6 O6 Na S2
	673.2719	5.0	7.4	28.5	50.5	C47 H38 O3 Na
	673.2719	5.0	7.4	15.5	17.4	C32 H42 N8 O3 Na S2
	673.2716	5.3	7.9	32.5	50.3	C45 H33 N6 O
	673.2824	-5.5	-8.2	19.5	23.8	C38 H42 N4 O4 Na S
	673.2712	5.7	8.5	19.5	25.3	C39 H42 N2 O5 Na S
	673.2711	5.8	8.6	26.5	64.5	C45 H41 N2 S2
	673.2828	-5.9	-8.8	32.5	49.4	C44 H33 N8
	673.2710	5.9	8.8	16.5	16.1	C31 H38 N8 O8 Na
	673.2709	6.0	8.9	23.5	24.5	C37 H37 N8 O3 S
	673.2831	-6.2	-9.2	28.5	49.5	C46 H38 N2 O2 Na
	673.2706	6.3	9.4	10.5	13.5	C31 H46 N4 O7 Na S2
	673.2835	-6.6	-9.8	17.5	24.2	C39 H45 O8 S

Figure S3:UPLC-MS Mass data: [natGa]MHLL1

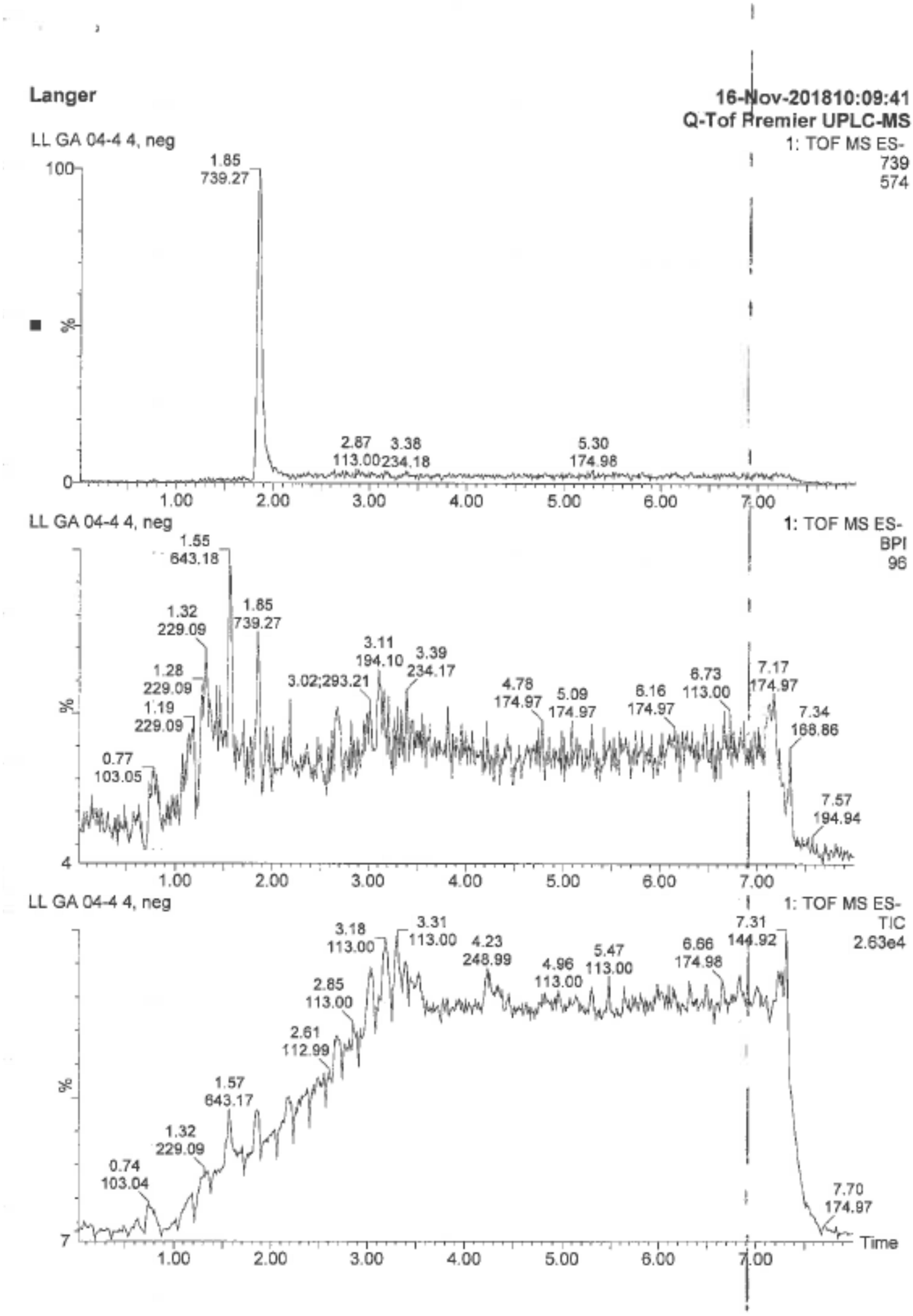

Elemental Composition Report

Single Mass Analysis

Tolerance = 20.0 PPM / DBE: min = -0.5, max = 50.0
Selected filters: None

Monoisotopic Mass, Even Electron Ions
7550 formula(e) evaluated with 162 results within limits (all results (up to 1000) for each mass)
Elements Used:
C: 0-55 H: 0-100 N: 0-9 O: 0-12 Na: 0-1 S: 0-1 Ga: 0-1

Langer Q-Tof Premier UPLC-MS 16-Nov-201810:09:41
LL GA 04-4 4, neg 197 (1.863) AM (Cen,4, 60.00, Ar,11000.0,554.26,0.55,LS 5) 1: TOF MS ES- 506

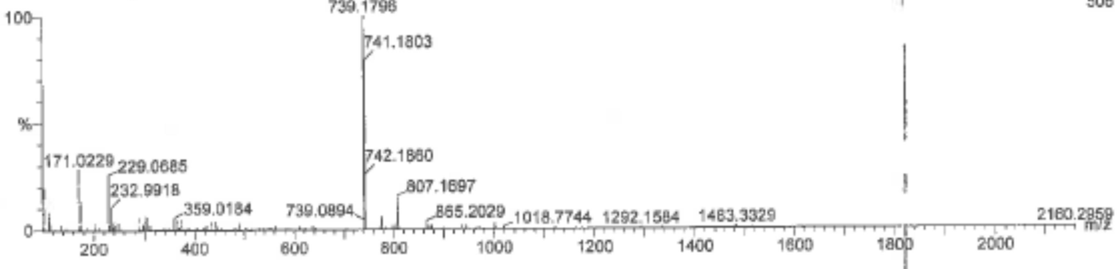

Minimum:				-0.5		
Maximum:		5.0	20.0	50.0		

Mass	Calc. Mass	mDa	PPM	DBE	i-FIT	Formula
739.1796	739.1796	0.0	0.0	25.5	1.5	C38 H34 N6 O6 Ga
	739.1797	-0.1	-0.1	28.5	5.9	C46 H38 O3 S Ga
	739.1798	-0.2	-0.3	21.5	1.5	C40 H39 O8 Na Ga
	739.1798	-0.2	-0.3	21.5	129.7	C34 H32 N6 O10 Na S
	739.1792	0.4	0.5	12.5	0.3	C32 H43 N2 O10 Na S Ga
	739.1791	0.5	0.7	25.5	141.9	C41 H32 O12 Na
	739.1791	0.5	0.7	32.5	122.8	C47 H31 O7 S
	739.1789	0.7	0.9	29.5	143.8	C39 H27 N6 O10
	739.1789	0.7	0.9	16.5	0.3	C30 H38 N8 O8 S Ga
	739.1804	-0.8	-1.1	37.5	124.9	C48 H27 N4 O3 S
	739.1805	-0.9	-1.2	17.5	0.0	C33 H39 N6 O6 Na S Ga
	739.1805	-0.9	-1.2	30.5	143.0	C42 H28 N4 O8 Na
	739.1786	1.0	1.4	43.5	140.6	C54 H24 N2 O Na
	739.1782	1.4	1.9	20.5	0.6	C37 H38 N2 O10 Ga
	739.1782	1.4	1.9	20.5	131.2	C31 H31 N8 O12 S
	739.1780	1.6	2.2	34.5	125.8	C46 H28 N4 O3 Na S
	739.1812	-1.6	-2.2	26.5	2.9	C41 H35 N4 O4 Na Ga
	739.1816	-2.0	-2.7	28.5	139.8	C43 H31 O12
	739.1776	2.0	2.7	11.5	1.2	C29 H42 N4 O12 S Ga
	739.1816	-2.0	-2.7	15.5	0.1	C34 H42 N2 O10 S Ga
	739.1818	-2.2	-3.0	35.5	144.3	C43 H24 N8 O4 Na
	739.1773	2.3	3.1	25.5	4.2	C44 H39 O3 Na S Ga
	739.1772	2.4	3.2	22.5	0.8	C36 H35 N6 O6 Na Ga
	739.1820	-2.4	-3.2	38.5	125.6	C51 H28 N2 O Na S
	739.1771	2.5	3.4	29.5	4.1	C42 H34 N6 O S Ga
	739.1822	-2.6	-3.5	24.5	127.3	C36 H31 N6 O10 S
	739.1822	-2.6	-3.5	24.5	2.6	C42 H38 O8 Ga
	739.1770	2.6	3.5	42.5	140.2	C51 H23 N4 O3
	739.1825	-2.9	-3.9	31.5	4.7	C42 H31 N8 Na Ga
	739.1825	-2.9	-3.9	20.5	125.5	C38 H36 O12 Na S
	739.1766	3.0	4.1	29.5	124.0	C45 H32 O7 Na S
	739.1765	3.1	4.2	13.5	1.2	C28 H39 N8 O8 Na S Ga
	739.1765	3.1	4.2	26.5	146.3	C37 H28 N6 O10 Na
	739.1764	3.2	4.3	33.5	125.6	C43 H27 N6 O5 S
	739.1764	3.2	4.3	33.5	9.1	C49 H34 O3 Ga
	739.1829	-3.3	-4.5	20.5	0.3	C35 H38 N6 O6 S Ga
	739.1829	-3.3	-4.5	33.5	141.2	C44 H27 N4 O8
	739.1830	-3.4	-4.6	13.5	1.2	C29 H39 N6 O11 Na Ga
	739.1832	-3.6	-4.9	16.5	0.4	C37 H43 O8 Na S Ga
	739.1758	3.8	5.1	17.5	134.5	C29 H32 N8 O12 Na S
	739.1758	3.8	5.1	17.5	0.3	C35 H39 N2 O10 Na Ga
	739.1757	3.9	5.3	24.5	2.6	C41 H38 N2 O5 S Ga

Figure S4: Radiochromatogram of [^{68}Ga]MHLL1

For all HPLC chromatograms following methods were used:

10% Methanol: 90% 70 mM phosphate buffer (pH = 2.5) to 50% Methanol: 50% 70 mM phosphate buffer (pH = 2.5) in 13 minutes leading to a radiotracer retention time of 11.75 minutes (column: Phenomenex Luna C18(2), 150x4.6 mm, 5 µM, 100 Å).

Figure interrupted due to page break in original

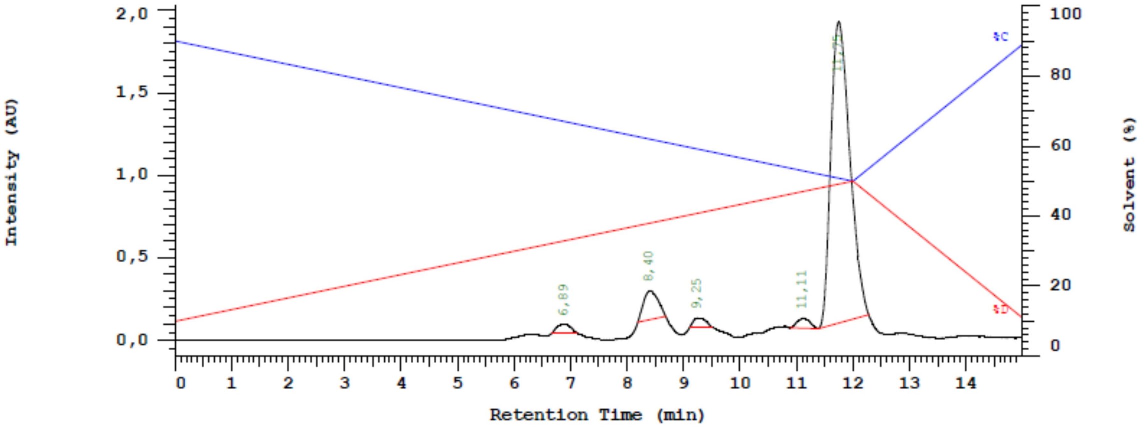

No.	RT	Area	Name	Conc 1	BC
1	6,89	434101		1,813	BB
2	8,40	1517564		6,336	BB
3	9,25	417535		1,743	BB
4	11,11	475353		1,985	BB
5	11,75	21105390		88,123	BB
		23949943		100,000	

Figure S5: Radiochromatogram from efflux studies (60 minutes)

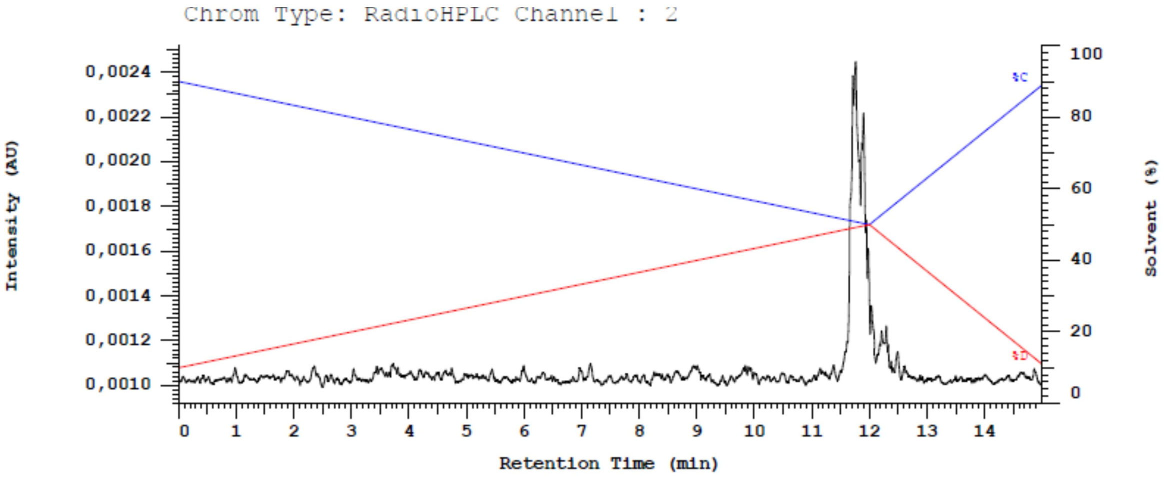

Figure S6: Radiochromatogram from biodistribution (urine) of [^{68}Ga]MHLL1 in healthy mice.

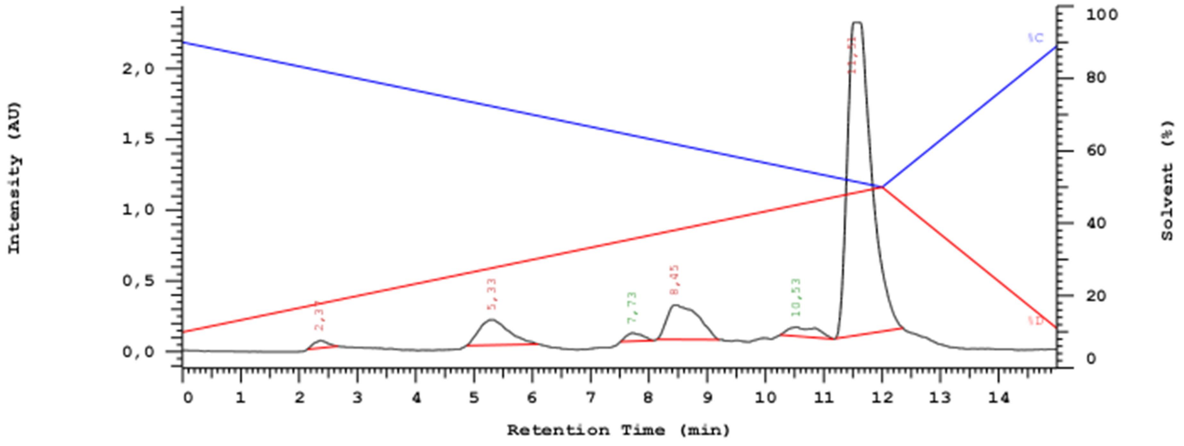

No.	RT	Area	Name	Conc 1	BC
1	2,37	425253		1,021	MC
2	5,33	3173267		7,621	MC
3	7,73	562212		1,350	BB
4	8,45	4568935		10,972	MC
5	10,53	1227007		2,947	BB
6	11,51	31684480		76,089	MC
		41641154		100,000	

Figure S7: Representative cardiac axis images of [^{68}Ga]MHLL1 (colourscale) and [^{18}F]FDG (greyscale) display accumulation of the fibroblast activation protein-targeted radiotracer in the non-viable infarct region. Blocking with excess unlabeled MHLL1 (1mg/kg) lowers accumulation in the infarct and remote myocardial regions without affecting the liver signal. SA, short axis; HLA, horizontal long axis.

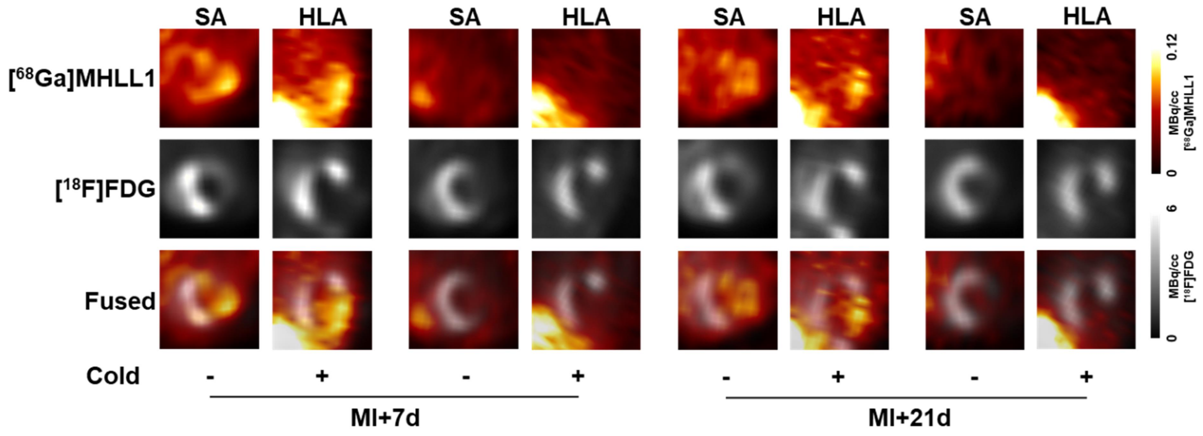

Figure S8: (A) Time activity curves for whole body distribution of [^{68}Ga]MHLL1 after myocardial infarction display rapid accumulation in liver and clearance through the renal system. Blood activity is reduced **(B)** Time activity curves of infarct and remote non-infarct myocardium display deviation between the regions from 15 – 60 min after tracer injection. Images display average activity concentration +/- standard deviation over the image acquisition for n=3 mice at each timepoint. The stable separation of the regions and the heart guided the selection of static imaging timepoint at 50 – 60 min after tracer injection.

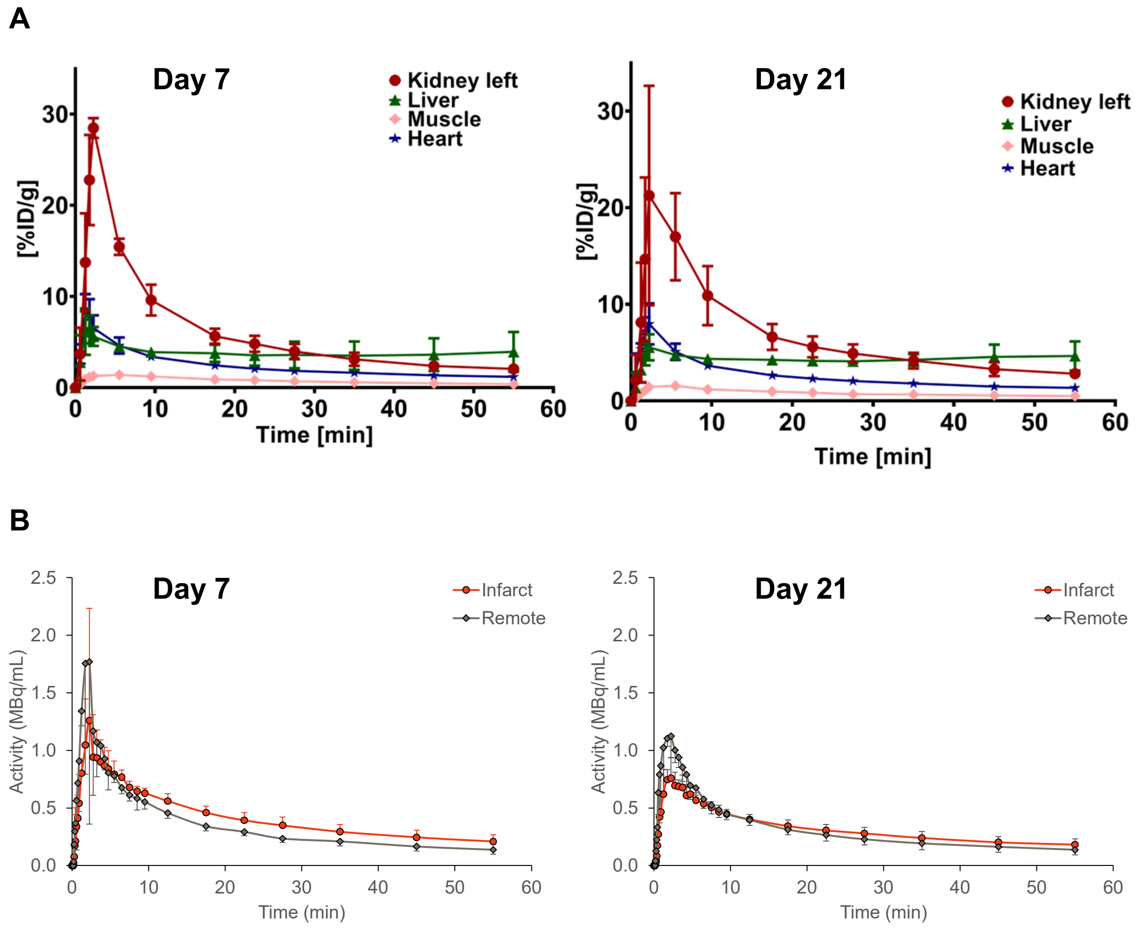

Supplement: Supplementary file 1 — Supplementary figures. [file thnov11p7755s1.pdf]
